# Open versus closed vitrification system of human oocytes and embryos: a systematic review and meta-analysis of embryologic and clinical outcomes

**DOI:** 10.1186/s12958-018-0440-0

**Published:** 2018-12-06

**Authors:** Hongcai Cai, Jean Damascene Niringiyumukiza, Yamin Li, Qiaohong Lai, Yinzhao Jia, Ping Su, Wenpei Xiang

**Affiliations:** 1 Family Planning Research Institute/Center of Reproductive Medicine, Tongji Medical College, Huazhong University of Science and Technology, Wuhan, 430030 Hubei China; 2 Center of Reproductive Medicine, Tongji Hospital, Tongji Medical College, Huazhong University of Science and Technology, Wuhan, 430030 Hubei China; 3 Department of General Surgery, Union Hospital, Tongji Medical College, Huazhong University of Science and Technology, Wuhan, 430030 Hubei China

**Keywords:** Vitrification, Cryopreservation, Oocyte, Blastocyst, Embryo, Meta-analysis

## Abstract

**Background:**

The objective of this study was to carry out a systematic review and meta-analysis of embryologic and clinical outcomes following open versus closed vitrification of human oocytes and embryos.

**Methods:**

An electronic literature search was conducted in main electronic databases up to June 30, 2018 using the following key terms: ‘oocyte’, ‘embryo’, ‘blastocyst’, ‘vitrification’, ‘cryopreservation’, ‘device’, ‘survival rate’, ‘pregnancy rate’, etc. A meta-analysis was performed using a random effect model to estimate the value of risk ratios (RRs) and 95% confidence interval (CI). Subgroup analyses and sensitivity analyses were carried out to further confirm the results.

**Results:**

Twelve **(**Eight prospective and four retrospective) studies comparing open versus closed vitrification of human oocytes or embryos were included. For prospective studies on oocytes, no evidence for a significant difference in cryosurvival rate (RR = 0.91, 95% CI: 0.80–1.03, *P* = 0.14; *n* = 2048) or clinical pregnancy rate (RR = 1.29, 95% CI: 0.80–2.06, *P* = 0.30; *n* = 150) was observed. Additionally, there were no significant differences between the two methods concerning secondary endpoints included positive βHCG rate, implantation rate, miscarriage rate, ongoing pregnancy rate, live birth rate, cancellation rate, babies born per transferred blastocysts, or multiple birth rate (*P* > 0.05). The results of the retrospective studies were similar as the prospective studies.

**Conclusions:**

It is still impossible to conclude that closed vitrification system could be a substitution for open system in human oocyte and embryo cryopreservation based on current evidence. Therefore, more well-designed prospective studies addressing these issues are still warranted.

## Background

Vitrification is one of the cryopreservation methods applied in the field of assisted reproductive technology (ART). With its high efficiency and consistency, vitrification is becoming the principal approach for cryopreservation of human oocytes and embryos, taking the place of traditional slow freezing in ART [1]. Several studies have proved that vitrification/warming is superior to slow-freezing/ thawing with regard to embryologic and clinical outcomes for oocytes, cleavage-stage embryos, and blastocysts [2–4], which have been further supported by a recent published systematic review and meta-analysis [5]. Hence, vitrification is becoming an extensively used approach for cryopreservation in reproductive centres all over the world, as has been recommended by the American Society for Reproductive Medicine [6].

However, there are still concerns about the application and promotion of this new technique, particularly regarding biosafety issues. Depending on whether there is direct contact with the liquid nitrogen (LN_2_), vitrification is commonly categorised into two types: ‘open’ and ‘closed’ system. While open vitrification reaches extreme high cooling rates due to direct contact with LN_2_, risks for potential cross-contamination and disease transmission mediated through LN_2_ increase when considering long-term cryopreservation. Alternatively, closed vitrification can avoid direct contact with LN_2_ by substituting the high concentration of cryoprotectant, thus influencing the efficiency of cooling [1]. As a consequence, relative studies have reported a decrease in cryosurvival rate which was attributed to closed vitrification [7, 8]. Currently, concerns about the long-term effects of vitrification on large-scale oocytes and embryos when using high concentrations of cryoprotectant, which is regarded as cytotoxic, still exist. Viral cross-contamination between human oocytes and/or embryos in containers for cryostorage has not been identified so far [9, 10]. However, environmental bacteria were identified in all the samples of LN_2_ collected from the oocyte/embryo containers, including open and closed devices, which could potentially do harm to the development of gametes and embryos [11]. Thus, it is still argued that the exposure of samples in open containers where environmental pathogens may exist can raise the contingent infectious danger for any IVF laboratory [12].

For the time being, however, results from different IVF laboratories around the world on the effects and safety of these two kinds of vitrification conflict. Regarding embryo cryopreservation, several studies reported comparable results between open and closed vitrification on survival rates after thawing [13–18]; while others demonstrated that closed vitrification outperformed the open one [19, 20]. For oocytes vitrification, some suggested that ‘open’ was better than ‘closed’ in post-thawing survival rates [7, 8, 21]; others found no statistical differences between these two methods [22, 23]. In addition, the rest of the embryologic and clinical outcomes differed from each other according to the studies mentioned above. Therefore, our study aims to compare the embryological and reproductive outcomes between open and closed vitrification for human oocytes and embryos (cleavage-stage embryo and blastocyst), by searching for relevant literatures through electronic databases in the past decades, thus attempting to provide evidence-based medical support for clinically infertile couples seeking fertility cryopreservation.

## Methods

### Search strategy

Studies were searched for according to the date of June 30, 2018, through several main electronic databases, including PubMed, MEDLINE, EMBASE, Cochrane Central Register of Controlled Trials (CENTRAL), Cochrane Libraries, World Health Organization (WHO) International Clinical Trials Registry Platform (ICTRP), Clinicaltrials.gov, and Current Controlled Trials to identify those which assessed embryological and reproductive outcomes by using oocytes and/or embryos vitrified by open or closed devices from women undergoing ART. The following search terms were applied, ‘oocyte’, ‘embryo’, ‘blastocyst’, ‘cleavage-stage embryo’, ‘vitrification’, ‘cryopreservation’, ‘cryosurvival rate’, ‘pregnancy rate’, and ‘live birth rate’, without language restrictions. These searches were limited to human studies. Additionally, relevant studies from the citation list of all retrieved publications and review articles were hand-searched. We contacted the corresponding authors for further information if the primary studies were inadequate for analysis. Our study was approved by the Institutional Review Board of Family Planning Research Institute of Tongji Medical College, Huazhong University of Science and Technology.

### Study selection and eligibility criteria

Two reviewers (HCC and JDN) performed an initial screening of all titles and abstracts independently. Studies were considered eligible if they (1) were prospective or retrospective studies, (2) compared open with closed vitrification as cryopreservation approaches, (3) used oocytes and/or embryos (cleavage and/or blastocyst stage) as participants, (4) used embryological (cyrosurvival rate as primary outcome, and fertilization rate, cleavage rate, good quality embryo rate or implantation rate, etc. as secondary outcomes) and/or reproductive outcomes (clinical pregnancy rate and/or live birth rate as primary outcome, and positive βHCG rate, cancellation rate, miscarriage rate, ongoing pregnancy rate, or multiple birth rate, etc. as secondary outcomes.) as outcomes of interest (at least one primary indicator was involved in a single study), (5) were conducted on humans. Given that there were at least 30 different carrier tools published at present [1], for convenience, we here defined ‘open’ devices as those with direct contact between the sample-containing medium and LN_2_, such as Cryotop (KITAZATO BIOPHARMA CO., LTD, JAPAN), Cryoloop (Hampton Research CO., LTD, USA), open pulled straw, etc.; while ‘closed’ devices were those without direct contact with LN_2_, for instance, Cryotip (Irvine Scientific, USA), Rapid-i (Vitrolife, Sweden), vitrisafe (IVF Distribution GmbH, Bregenz, Austria), closed pulled straw, and so on. Full texts were retrieved for further information if they satisfied the selection criteria. Studies conducted only on animals, without at least one primary indicator, and were not intact gametes or embryos although with elaborate design and of good quality, otherwise, were excluded. Review articles, conference abstracts, unpublished data, and cases reports were also considered as ineligible. Any discrepancy was resolved after hosting discussions with all authors until a consensus was reached.

### Data extraction and methodological quality evaluation

A data extraction sheet was well designed beforehand to collect relevant information which included: demography (first author name, year, country, title of the study, journal, study period, number of patients/oocytes/embryos included, characteristics of the study participants and funding sources, conflicts of interest), methodology (study design, method of randomization, quality score), procedure (inclusion/exclusion criteria, ovarian stimulation and ovulation triggering protocols, type of cryo-carrier and cryoprotectant used, type of fertilization, stage at collection and transfer, cooling and warming rate, number of embryos transferred), and outcome data (relative embryological and reproductive outcome measures as previously described). Two independent authors extracted data and carefully assessed the quality of each study. In cases of disagreement, a consensus was reached after discussion between the two authors. To avoid inclusion of duplicate or overlapping samples, we meticulously compared the original areas of the studies. In the event of data overlapping, we included the latest version with the largest number of cases or the values of risk ratio (RR) and 95% confidence intervals (CIs) that were adjusted. We used ROBINS-I tool for assessing the quality of all the included studies [24].

### Data synthesis and meta-analysis

Meta-analyses of the included studies were employed to estimate the pooled RR value and 95% CIs of all the outcome measures. Heterogeneity of the studies was assessed using a *Q* test and an *I*^*2*^ index. A random effect model was used to estimate the value of risk ratios (RRs) and 95% confidence interval (CI). Subgroup analyses were carried out according to the study location, study quality, and risks of bias, with the purpose of investigating substantial heterogeneity that might affect the cumulative evidence. When we detected substantial heterogeneity, a sensitivity analysis was then performed to explore possible explanations and further confirm the consistency of the outcomes. Statistical heterogeneity was taken into account when interpreting the results. An alpha value of 0.05 under a two-sided test was considered as statistically significant. The existence of publication bias was evaluated by establishing funnel plots as well as by performing Begg’s [25] and Egger’s test [26]. We used the Grading of Recommendations Assessment, Development and Evaluation (GRADE) system (study limitations, consistency of effect, imprecision, indirectness, and publication bias) to assess the quality of the evidence for all outcomes [27].

The meta-analysis and constructions of forest and funnel plots were performed with Review Manager Software (Version 5.3 for Mac; Copenhagen: The Nordic Cochrane Centre, The Cochrane Collaboration, 2014). Stata/SE (version 12.0 for Mac) was utilised for Begg’s and Egger’s tests.

## Results

### Identification of literature

**Fig. 1:**
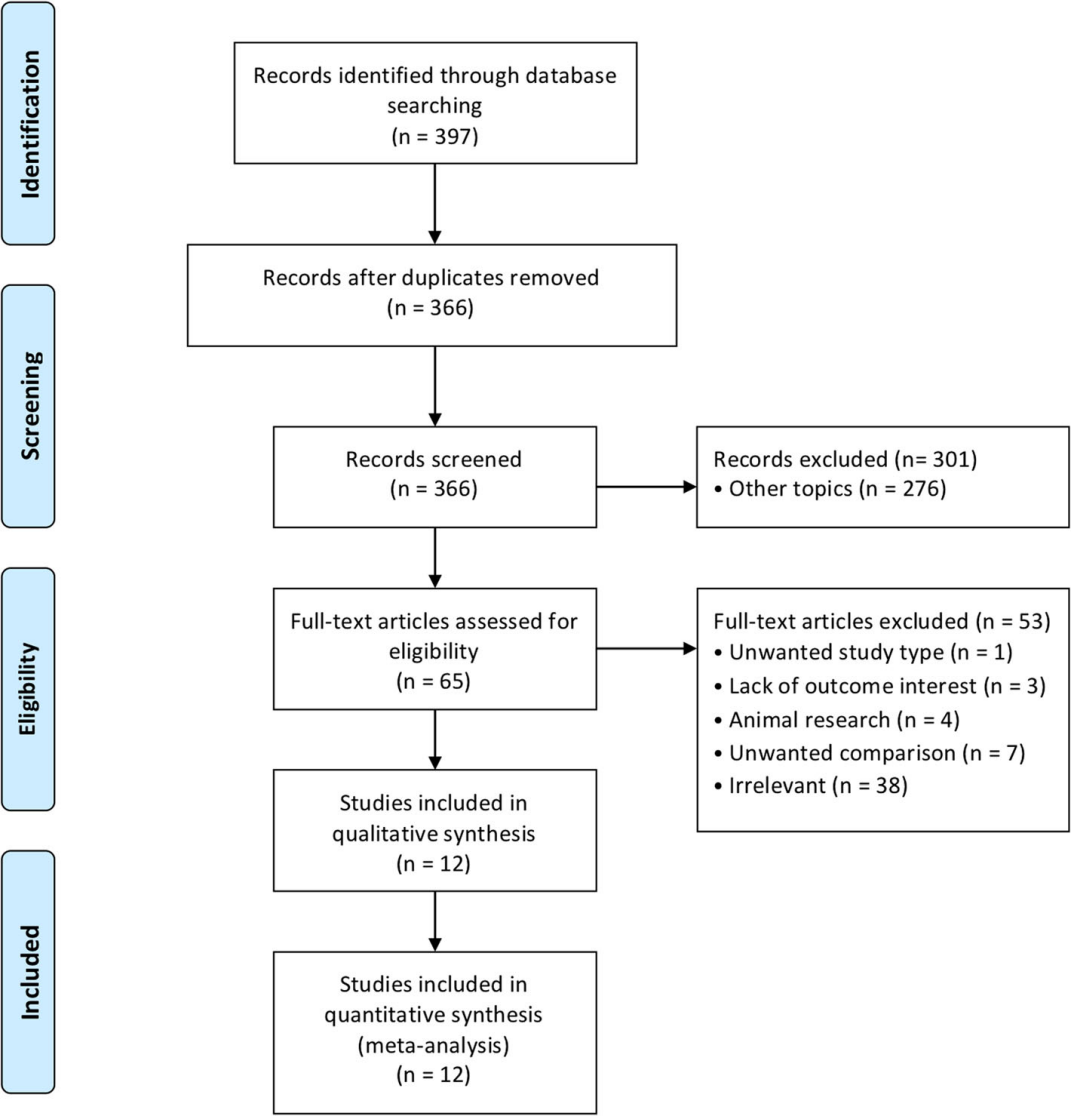
Flow diagram for a systematic review and meta-analysis comparing open versus closed vitrification of oocytes and embryos in ART

All studies comparing open versus closed vitrification of oocytes and/or embryos on embryologic and clinical reproductive outcomes were considered eligible in this systematic review and meta-analysis. The electronic search retrieved 397 records initially. Of these, 31 studies were excluded as duplicates and 366 records remained and were subsequently screened based on their title and abstract. Of these, 301 were consequently excluded because of obvious unrelated researches. The full texts of the remaining 65 articles were retrieved to be assessed for eligibility. Of these, 53 studies were excluded and the reasons for exclusion were as followed: not the type of comparison; lack of primary outcomes; animal research; review article. In total, 12 articles were included in the quantitative synthesis. The flow diagram of the selection procedure is presented in Fig. 1.

### Characteristics of the included studies and quality assessment

There were 12 studies (eight prospective and four retrospective studies) included in quantitative analysis. These consisted of five studies on oocytes vitrification and nine studies on embryo vitrification, among which the data of two studies extracted from the oocytes studies [7, 22]. In total, 2577 oocytes (1330 for open and 1247 for closed vitrification, respectively) and 3640 embryos (2024 for open and 1616 for closed vitrification, respectively; data from one study [22] was not counted because the author didn’t mention the number of embryos thawed in the study) were included for quantitative analysis. The language of the included studies was English, except for one in French [21]. The participants in these studies mainly came from Europe, North America, and East Asia. The basic characteristics of the selected literatures are listed on Table 1. Results of the methodological quality assessment showed that 3 of the prospective studies were of low risk of bias [8, 17, 22], 2 were moderate [13, 16] and 3 were of serious risk of bias [20, 21, 23]. The results of the quality assessment of the included retrospective studies were shown in Table 1.

**Table 1:** Basic information of the included studies comparing open versus closed vitrification on oocytes and embryos

Authors, Country (Year)	Study period	Study type	Participant	No. of cycles and oocytes/embryos thawed		Outcomes	Funding	Overall quality assessment
				Study (Closed)	Control (Open)			
Kuwayama et al. (2005), Japan	NM	Prospective	Embryo	88 embryos	227 embryos	CsR; CPR; LBR	None declared	Moderate risk
Chen et al. (2013), China	2010.04–2012.05	Retrospective	Embryo	226 cycles; 308 embryos	106 cycles; 150 embryos	CsR; IR; CaR; CPR; OPR; MR; LBR; BB; MR	Yes	Low risk
Panagiotidis et al. (2013), Greece	2009.06–2011.09	Prospective	Embryo	224 cycles; 513 embryos	208 cycles; 492 embryos	CsR; PβR; IR; CaR; CPR; OPR; MR; LBR; BB; MR	None declared	Low risk
Desai et al. (2013), America	2011.01–2012.08	Retrospective	Embryo	95 cycles; 184 embryos	161 cycles; 302 embryos	CsR; IR; CaR; CPR; OPR; MR; LBR; BB; MR	None declared	Low risk
Hashimoto et al. (2013), Japan	2011.07–2012.09	Prospective	Embryo	100 embryos	163 embryos	CsR; IR; OPR	Yes	Moderate risk
Roy et al., Australia (2014)	NM	Prospective	Embryo	23 embryos	13 embryos	CsR; OPR	Yes	Serious risk
Iwahata et al. (2015), Japan	2011.11–2013.12	Retrospective	Embryo	302 cycles; 313 embryos	539 cycles; 561 embryos	CsR; IR; CPR; MR; LBR; MR	None declared	Low risk
Paffoni et al. (2011), Italy	2007.06–2009.04	Retrospective	Oocyte/embryo	51 cycles; 261 MII oocytes/87 embryos	53 cycles; 268 MII oocytes/116 embryos	CsR; IR; FR; ClR; GQER; CPR; LBR	None declared	Low risk
Papatheodorou et al. (2013), Greece	2007.02–2010.12	Prospective	Oocyte	598 MII oocytes	608 MII oocytes	CsR; FR; ClR; GQER; PβR; IR; CPR; MR; OPR; LBR	None declared	Low risk
De Munck et al. (2016), Belgium	2014.01–2015.07	Prospective	Oocyte/embryo	253 MII oocytes	257 MII oocytes	CsR; FR; GQER; PβR; MR; OPR	No funding	Low risk
Gook et al. (2016), Australia	2012.06–2015.06	Prospective	Oocyte	39 MII oocytes	79 MII oocytes	CsR	None declared	Serious risk
Sarandi et al. (2016), France	2014.11–2015.05	Prospective	Oocyte	96 GV/MI oocytes	118 GV/MI oocytes	CsR	None declared	Serious risk

Notes: The ROBINS-I tool was used for quality assessment of observational studies. *BB* babies born per transferred blastocysts, *CaR* cancellation rate, *CBS* Cryo Bio System, *ClR* cleavage rate, *CPR* clinical pregnancy rate, *CsR* cryosurvival rate, *FR* fertilization rate, *GQER* good quality embryo rate, *HSV* high security straws, *IR* implantation rate, *LBR*: live birth rate, *MBR* multiple birth rate, *MR* miscarriage rate, *NM* not mentioned, *OPR* ongoing pregnancy rate, *PβR* positive βHCG rate

### Publication bias evaluation

Allowing for the difficulty of detecting and correcting for publication bias and other reporting biases, we tried to minimise their potential influence by ensuring a comprehensive search for eligible studies and by paying attention to the duplicated data. Results of Begg’s and Egger’s tests revealed that no significant publication biases were shown among the included studies, as presented in Tables 2 and 3.

**Table 2:** Results of meta-analyses and test of publication bias on oocytes

Outcomes	Study type	No. of studies	Events/Total		RR, 95%CI, *P*	Test of Heterogeneity		Tests of Publication Bias	
			Closed	Open	Random	*I*^2^ (%)	*P*	Begg’s *P*	Egger’s *P*
Cryosurvival rate	Prospective	4	830/986	967/1062	0.91, [0.80, 1.03], 0.14	91%	0.00	1.000	0.921
	Retrospective	1	222/268	151/261	1.43, [1.27, 1.61], < 0.01	NA	NA	NA	NA
Fertilization rate	Prospective	2	585/733	594/784	1.02, [0.83, 1.24], 0.88	92%	0.00	1.000	NA
	Retrospective	1	116/159	87/151	1.27, [1.07, 1.50], < 0.01	NA	NA	NA	NA
Good quality embryo rate	Prospective	2	229/561	240/566	0.97, [0.84, 1.11], 0.62	0%	0.96	1.000	NA
	Retrospective	1	62/112	30/87	1.61, [1.15, 2.24], < 0.01	NA	NA	NA	NA
Positive βHCG rate	Prospective	2	45/109	47/125	1.09, [0.75, 1.59], 0.64	22%	0.26	1.000	NA
Miscarriage rate	Prospective	2	4/38	8/39	0.59, [0.20, 1.79], 0.35	0%	0.84	1.000	NA
Ongoing pregnancy rate	Prospective	2	38/125	27/109	1.24, [0.81, 1.89], 0.32	0%	0.45	1.000	NA
Clinical pregnancy rate	Prospective	1	27/75	21/75	1.29, [0.80, 2.06], 0.30	NA	NA	NA	NA
	Retrospective	1	14/53	4/51	3.37, [1.19, 9.55], 0.02	NA	NA	NA	NA
Live birth rate	Prospective	1	27/75	18/75	1.50, [0.91, 2.48], 0.11	NA	NA	NA	NA
	Retrospective	1	11/53	3/51	3.53, [1.04, 11.92], 0.04	NA	NA	NA	NA

*NA* not applicable, *RR* risk ratio

**Table 3:** Results of meta-analyses and test of publication bias on embryos

Outcomes	Study type	No. of studies	Events/Total		RR, 95%CI, *P*	Test of Heterogeneity		Tests of Publication Bias	
			Closed	Open	Random	*I*^2^ (%)	*P*	Begg’s *P*	Egger’s *P*
Cryosurvival rate	Prospective	4	621/724	803/895	0.98, [0.96, 1.01], 0.29	0%	0.42	0.734	0.612
	Retrospective	3	770/805	959/1013	1.00, [0.97, 1.03], 0.88	58%	0.09	1.000	0.940
Positive βHCG rate	Prospective	2	115/222	114/211	0.96, [0.80, 1.14], 0.64	0%	0.77	1.000	NA
Implantation rate	Prospective	3	160/535	196/590	0.98, [0.83, 1.15], 0.80	0%	0.93	1.000	0.798
	Retrospective	4	333/839	444/1071	0.97, [0.81, 1.17], 0.76	53%	0.09	0.308	0.805
Cancellation rate	Retrospective	2	9/321	9/267	0.75, [0.18, 3.04], 0.69	30%	0.23	1.000	NA
Clinical pregnancy rate	Prospective	3	134/304	226/432	0.89, [0.76, 1.05], 0.17	0%	0.91	1.000	0.613
	Retrospective	4	280/513	374/577	0.96, [0.79, 1.17], 0.69	61%	0.05	0.734	0.790
Ongoing pregnancy rate	Prospective	3	133/319	160/369	0.98, [0.82, 1.16], 0.79	0%	1.00	1.000	0.682
	Retrospective	2	114/312	105/258	0.96, [0.71, 1.29], 0.77	50%	0.16	1.000	NA
Miscarriage rate	Prospective	2	4/93	8/94	0.60, [0.11, 3.31], 0.56	43%	0.18	1.000	NA
	Retrospective	3	61/276	76/360	1.07 [0.79, 1.46], 0.66	0%	0.71	1.000	0.337
Live birth rate	Prospective	2	123/287	193/415	0.96, [0.81, 1.15], 0.69	0%	0.71	1.000	NA
	Retrospective	4	217/513	313/577	0.88 [0.71, 1.10], 0.26	48%	0.12	0.308	0.163
Babies born/ transferred blastocysts	Retrospective	2	114/438	124/420	0.82 [0.65, 1.02], 0.09	0%	0.48	1.000	NA
Multiple birth rate	Retrospective	3	30/293	21/357	0.95 [0.41, 2.24], 0.92	52%	0.13	1.000	0.831

*NA* not applicable, *RR* risk ratio

### Open versus closed vitrification on oocytes: Embryologic and clinical outcomes

Five studies concerning oocytes vitrification were included for meta-analysis (See summary in Table 2). No evidence for a difference was found in regards to the outcome of the cryosurvival rate when comparing closed and open vitrification on oocytes, respectively (RR = 0.91, 95% CI: 0.80–1.03, *P* = 0.14; *n* = 2048, 986 versus 1062 oocytes; *I*^2^ = 91%, low quality evidence). There was no significant difference regarding the clinical pregnancy rate (RR = 1.29, 95% CI: 0.80–2.06, *P* = 0.30; *n* = 150, 75 versus 75 transferred cycles). Similarly, no differences were identified between the groups regarding fertilization rate (RR = 1.02, 95% CI: 0.83–1.24, *P* = 0.88; *n* = 1517, 733 versus 784 oocytes;*I* ^2^ = 92%, low quality evidence), good quality embryo rate (RR = 0.97, 95% CI: 0.84–1.11, *P* = 0.62; *n* = 1127, 561 versus 566 embryos; *I* ^2^ = 0%, moderate quality evidence), positive βHCG rate (RR = 1.09, 95% CI: 0.75–1.59, *P* = 0.64; *n* = 234, 109 versus 125 transferred cycles; *I*^2^ = 22%, moderate quality evidence), miscarriage rate (RR = 0.59, 95% CI: 0.20–1.79, *P* = 0.35; *n* = 77, 38 versus 39 clinical pregnancy cycles; *I*^2^ = 0%, moderate quality evidence), ongoing pregnancy rate (RR = 1.24, 95% CI: 0.81–1.89, *P* = 0.32; n = 234, 125 versus 109 transferred cycles; *I*^2^ = 0%, moderate quality evidence), or live birth rate (RR = 1.50, 95% CI: 0.91–2.48, *P* = 0.11; *n* = 150, 75 versus 75 clinical pregnancy cycles). Sensitivity analyses were performed by excluding the study with the largest weight, smallest weight, or highest heterogeneity, and ended up with semblable conclusions.

### Open versus closed vitrification on embryos: Embryologic and clinical outcomes

A total of nine studies (five prospective and four retrospective studies) on embryos vitrification were adopted for quantitative analysis (See summary in Table 3). Four prospective studies on embryo cryopreservation (closed versus open) demonstrated no difference in cryosurvival rate (RR = 0.98, 95% CI: 0.96–1.01, *P* = 0.29; *n* = 1619, 724 versus 895 oocytes; *I*^2^ = 0%, moderate quality evidence) and findings were consistent with three retrospective studies (RR = 1.00, 95% CI: 0.97–1.03, *P* = 0.88; *n* = 1818, 805 versus 1013 oocytes; *I*^2^ = 58%). There was no difference in clinical pregnancy rate (RR = 0.89, 95% CI: 0.76–1.05, *P* = 0.17; *n* = 736, 304 versus 432 transferred cycles; *I*^2^ = 0%, moderate quality evidence), when comparing closed to open vitrification, respectively, which was also supported by four relevant retrospective studies (RR = 0.96, 95% CI: 0.79–1.17, *P* = 0.69; *n* = 1090, 513 versus 577 oocytes; *I*^2^ = 58%). Additionally, no evidence for differences in positive βHCG rate (RR = 0.96, 95% CI: 0.80–1.14, *P* = 0.63; *n* = 433, 222 versus 211 transferred cycles; *I*^2^ = 0%, moderate quality evidence), implantation rate (RR = 0.97, 95% CI: 0.82–1.15, *P* = 0.74; *n* = 1125, 535 versus 590 transferred embryos; *I*^2^ = 0%, moderate quality evidence), miscarriage rate (RR = 0.48, 95% CI: 0.15–1.54, *P* = 0.22; *n* = 187, 93 versus 94 clinical pregnancy cycles; *I*^2^ = 43%, moderate quality evidence), ongoing pregnancy rate (RR = 0.98, 95% CI: 0.82–1.16, *P* = 0.79; *n* = 688, 319 versus 369 transferred cycles; *I*^2^ = 0%, moderate quality evidence), live birth rate (RR = 0.97, 95% CI: 0.81–1.15, *P* = 0.70; *n* = 702, 287 versus 415 transferred cycles; *I* ^2^ = 0%, moderate quality evidence), cancellation rate (RR = 0.75, 95% CI: 0.26–2.14, *P* = 0.59; *n* = 588, 321 versus 267 transferred cycles; *I*^2^ = 30%, moderate quality evidence), babies born per transferred blastocysts (RR = 0.82, 95% CI: 0.66–1.03, *P* = 0.08; *n* = 858, 438 versus 420 transferred embryos; *I*^2^ = 0%, moderate quality evidence), and multiple birth rate (RR = 0.95, 95% CI: 0.41–2.24, *P* = 0.92; *n* = 650, 293 versus 357 live birth cycles; *I*^2^ = 52%, moderate quality evidence) was shown. These findings were further confirmed by relevant retrospective studies and sensitivity analyses (Table 3).

### Subgroup analysis

We planned the following subgroup analyses for the outcomes with high heterogeneity, such as cryosurvival rates, implantation rate, clinical pregnancy rate, and multiple birth rate, according to the differences of the original areas, study quality, and risks of bias, which also displayed no significant changes in related embryologic or clinical outcomes.

## Discussion

In this systematic review and meta-analysis, we compared open versus closed vitrification for oocytes and embryos cryopreservation regarding post-thaw survival rate, clinical pregnancy rate, and other embryologic and clinical reproductive outcomes. We found that closed vitrification systems could achieve sound embryologic outcomes as ‘open’ ones for both human oocytes and embryos cryopreservation, although substantial heterogeneity existed in some of the outcomes. For more accurate findings, further studies should be performed to ensure sufficient data analysis.

Recently, two systematic reviews reported the reproductive outcomes of human oocytes and embryos comparing open versus closed vitrification [28, 29]. One study suggested that it was not yet possible to conclude that closed vitrification is an aseptic alternative to open vitrification in human mature oocyte cryopreservation. In this study, only four articles concerning cyrosurvival rate after vitrification were included, of which the strength of evidence was low [29]. The other study regarding embryo vitrfication concluded with similar results on cryosurvival rates, implantation rates, clinical pregnancy rates and live birth rates, where seven articles were included. Although there was no significant difference between these two methods, the tendency of lower live birth rates with closed vitrification than with open vitrification could be clearly identified [28]. Our study was consistent with the previous conclusions, although low to moderate levels of evidence were achieved. This is probably due to the limited numbers of studies included and the variability in quality. Moreover, the high heterogeneity across the studies could be considered a limitation of the study.

Several factors, including the cooling and warming rates, the concentration of cryoprotectants, the solution volume, etc., should be taken into account when considering the effects of vitrification on human oocytes and embryos. Vitrification in cryobiology is essentially designed to eliminate ice formation in the medium containing the sample during the whole procedure (cooling, storage, and warming), which can be achieved by accelerating the cooling and warming rates, as well as increasing the concentration of cryoprotectants [30–32]. The high cooling and warming rates may help to alleviate chilling injury [33], while highly concentrated cryoprotectants may cause toxic and osmotic injury [34]. For this reason, the use of the smallest solution volume and the highest temperature conductivity between the sample-containing medium and the cooling or warming agent is required to achieve the highest cooling and warming rates [35]. Contrary to common beliefs, it has been proved that the intracellular concentration of cryoprotectant in vitrified embryos is lower than after slow freezing, although the solutions used in vitrification contain higher concentrations of cryoprotectants [36]. According to our results, the cooling rate of these closed systems was lower compared to open ones, whereas the warming rate was nearly the same as that of an open system. Therefore, outcome measures of closed systems with lower cooing rates may be more easily affected in some extent, particularly with respect to oocytes, mainly due to its small surface: volume ratio [37]. To some extent, this may partially explain the reason that the observed cryosurvival rates of oocytes in closed systems exhibited a downregulated trend than the open groups in most of the studies included, although the overall survival rates between both groups are not statistically different. Otherwise, such differences were more ambiguous among embryos after vitrification, with regard to embryologic and reproductive outcomes as presented in our systematic review.

Vitrification in cryobiology is essentially designed to eliminate ice formation in the medium containing the sample during the whole procedure (cooling, storage, and warming), which can be achieved by accelerating the cooling and warming rates, as well as increasing the concentration of cryoprotectants [30–32]. The high cooling and warming rates may help to alleviate chilling injury [33], while highly concentrated cryoprotectants may cause toxic and osmotic injury [34]. For this reason, the use of the smallest solution volume and the highest temperature conductivity between the sample-containing medium and the cooling or warming agent is required to achieve the highest cooling and warming rates [35]. Contrary to common beliefs, it has been proved that the intracellular concentration of cryoprotectant in vitrified embryos is lower than after slow freezing, although the solutions used in vitrification contain higher concentrations of cryoprotectants [36]. Due to the lack of data on the cooling and warming rates of vitrification included in this systematic review, subgroup analyses were unable to performed, so as to find out their effect on reproductive outcomes. Nonetheless, on the whole, the cooling rate of these closed systems was lower compared to open ones, whereas the warming rate was nearly the same as that of an open system. Therefore, outcome measures of closed systems with lower cooing rates may be more easily affected in some extent, particularly with respect to oocytes, mainly due to its small surface: volume ratio [37]. To some extent, this may partially explain the reason that the observed cryosurvival rates of oocytes in closed systems exhibited a downregulated trend than the open groups in most of the studies included, although the overall survival rates between both groups are not statistically different. Otherwise, such differences were more ambiguous among embryos after vitrification, with regard to embryologic and reproductive outcomes as presented in our systematic review.

Vitrification has been widely used for cryopreservation during the past decade by virtue of better outcomes when compared to traditional slow freezing. Nevertheless, closed vitrification, without the risk of biosafety, which also achieves sound embryological outcomes as open one does, should attract a lot of attention in the future. In this case, the efficiency and consistency of this method for mammalian oocytes and embryos could still be promoted by optimizing each step of the process, which could be widely applied to future clinical practice.

Drawbacks inherent to the quality and quantity of the included studies, in particular, some important outcomes such as clinical pregnancy rate, live birth rate on embryos, with only one study involving limited sample size incorporated into quantitative analysis, have weakened the strength of available evidence. In addition, few to no data are available for synthetic analysis on the safety aspects inherent to offspring generation derived from frozen-thawed gametes, given that convincing evidence is sparse, until recently a study reported that neonatal outcomes were not different after transfer of vitrified blastocysts compared with cleavage-stage embryos [38]. Another weakness in our systematic review was that we did not analyse studies that compared open or closed vitrified oocytes/embryos with fresh oocytes/embryos. Thus, more strong evidence is needed to better understand the real effect and safety that vitrification could achieve.

The optimal embryological and clinical outcomes of oocytes/embryos achieved with the use of vitrification over the last decade have important clinical implications, for instance, enhancing the cumulative live birth rate per oocyte retrieval cycle, extending time for embryo evaluation, enabling egg banking for donation and/or for oocyte accumulation, etc., which together allow a personalised approach in the care of different populations for medical or non-medical indications [5, 10, 39, 40]. Nowadays, due to the avoidance of direct contact with LN_2_ and consequently, lowering the cross-contamination and disease transmission risks for long-term cryopreservation, closed vitrification has become more popular and widely used in IVF laboratories across the world [41].

## Conclusions

In conclusion, closed vitrification system is still unable to be an aseptic alternative for open system when considering human oocyte and embryo cryopreservation based on current evidence. More large-scale studies with consolidated criteria and delicate design are needed to further evaluate the efficiency and biosafety of vitrification for human oocytes and embryos, especially focusing on the oocyte quality of older sub-infertile/infertile patients, as well as prolonging the length of follow-ups for offspring. Furthermore, it’s necessary to keep on exploring novel cryoprotectant with low cytotoxicity and high efficiency, accelerating the cooling rate, and simplifying the procedure in all efforts to improve the technique of vitrification for ART. Given that the risk of biosafety still remains, otherwise, it is advocated that a rigorous process of standardization for vitrification should be placed on the agenda.

## Abbreviations

ART: Assisted reproductive technology
CI: Confidence interval
LN_2_: Liquid nitrogen
RR: risk ratio
